# Heat and Light Stability of Pumpkin-Based Carotenoids in a Photosensitive Food: A Carotenoid-Coloured Beverage

**DOI:** 10.3390/foods11030485

**Published:** 2022-02-07

**Authors:** Sharmaine Atencio, Sarah H. E. Verkempinck, Kai Reineke, Marc Hendrickx, Ann Van Loey

**Affiliations:** 1 Laboratory of Food Technology, Department of Microbial and Molecular Systems, KU Leuven, Kasteelpark Arenberg 22, 3001 Leuven, Belgium; sharmaine.atencio@student.kuleuven.be (S.A.); sarah.verkempinck@kuleuven.be (S.H.E.V.); marceg.hendrickx@kuleuven.be (M.H.); 2 GNT Europa GmbH, Kackertstrasse 22, 52072 Aachen, Germany; kreineke@gnt-group.com

**Keywords:** carotenoids, degradation, kinetics, ascorbic acid, pumpkins, light, oxidation

## Abstract

This study aimed to evaluate carotenoid degradation kinetics in a beverage coloured with pumpkin juice concentrate during storage at dark and illuminated conditions at four temperatures (10, 20, 35 and 45 °C). Carotenoids were quantified by HPLC-DAD, and kinetic parameters for carotenoid degradation were estimated by one-step nonlinear regression analysis. During dark storage, degradation kinetics was modelled by fractional conversion (all-trans-β-carotene) and zero-order equations (all-trans-antheraxanthin, all-trans-lutein, all-trans-violaxanthin and all-trans-neoxanthin). Storage of samples in a climatic chamber with intense light intensity (1875–3000 lux) accelerated the carotenoid losses. At illuminated conditions, degradation followed a first-order (all-trans-lutein, all-trans-violaxanthin and all-trans-neoxanthin) and fractional conversion model (all-trans-β-carotene and all-trans-antheraxanthin). Carotenoid degradation followed an Arrhenius temperature-dependency, with $E_{a}$ values lower than 50 kJ/mol. Degradation was shown to be mainly by oxidative reactions. Packaging under minimal oxygen conditions, use of antioxidants (e.g., ascorbic acid), and proper choice of light sources at retail shelves may be considered to optimize the pigment retention in a carotenoid-coloured beverage during storage.

## 1. Introduction

Recent consumer concerns about the safety of artificial colourants have led to an increased use of natural colourants in food products, such as carotenoid-rich colouring concentrates from pumpkins [1,2]. Carotenoids are natural pigments responsible for the yellow, orange, and red colour of many fruits, vegetables, algae, and bacteria. They are lipid-soluble isoprenoid compounds with a C40 carbon skeleton from which all different types are derived. Their distinctive colour is provided by the conjugated double-bond system constituting the light-absorbing chromophores [3]. The tetraterpenoid structure of carotenoids creates an electron-rich environment which promotes their antioxidant effect, but which simultaneously makes them highly reactive. Carotenoids can undergo two types of changes, degradation and trans-cis isomerisation, affecting both their colour intensity and biological activities [4]. The different mechanisms of carotenoid degradation and isomerisation reactions were comprehensively reviewed by Boon et al. (2010) and Pénicaud et al. (2011) [5,6]. Oxidation has been reported as the major cause of carotenoid degradation, leading to products such as short-chain molecules, epoxides, and apocarotenoids, as well as to trans-cis isomerisation [7].

Carotenoids have been extensively studied in several model systems and plant matrices, revealing different factors affecting their stability, such as carotenoid type and physical form, oxygen concentration, presence of metals, exposure to light, severity of heat treatment, food matrix, and storage temperature [8,9,10,11,12,13,14]. In contrast, studies describing the chemical stability of carotenoid-rich colouring concentrates in food applications are rather limited, although this information would be industrially relevant for predicting carotenoid losses and colour changes in coloured food products throughout their shelf-life. For instance, since processing of yellow- or orange-coloured beverages results in substantial colour loss, additional carotenoids are often added for colour preservation [2]. These products are then packaged in clear bottles and exposed to natural and artificial lights during retail display, often deteriorating the photosensitive carotenoids [13]. However, besides processing parameters, little information is available on the effect of packaging and storage conditions on the changes of these carotenoid-containing beverages, aiming to minimize undesirable colour changes due to carotenoid degradation. Additionally, to minimize carotenoid degradation, it is common practice to incorporate antioxidants such as water-soluble ascorbic acid [15]. Direct oxidative protection of carotenoids by exogenous ascorbic acid has been previously reported [16,17]. However, a contrasting observation was reported by Meléndez–Martínez et al. (2009) [18] for orange juice carotenoids, wherein samples with externally added ascorbic acid had significantly higher losses of xanthophylls compared to the non-fortified samples. Nonetheless, to the best of our knowledge, the reported influence of exogenous ascorbic acid in carotenoid stability kinetics in a carotenoid-coloured food product during storage has been limited. Kinetic studies of carotenoid losses in coloured food matrices are important in the context of predicting carotenoid content changes during processing and storage, which in turn are useful in developing products with an attractive colour.

Overall, this study aimed to study the degradation kinetics of the main carotenoids in a pumpkin colouring concentrate in a beverage application. The effects of storage temperature (10, 20, 35 and 45 °C) and illumination (dark vs. illuminated) were considered. This information is fundamental in predicting carotenoid losses and its relation to shelf-life of the beverage product during storage especially at retail conditions. Our investigation was further extended to confirm if ascorbic acid fortification of the beverage samples had a protective effect on the pigment stability, thereby investigating if oxidation was the main cause of carotenoid degradation in the product.

## 2. Materials and Methods

### 2.1. Materials

Pumpkin (*Cucurbita maxima Dueschne)* colouring concentrate, provided by GNT International B.V. (Mierlo, The Netherlands), was employed as carotenoid-based natural colourant. According to the manufacturer, the industrial production of the concentrate involved physical manufacturing processes (i.e., mechanical and aqueous enzymatic treatments), followed by concentration and pasteurisation. In the whole process, water was used as the sole processing medium.

### 2.2. Beverage Preparation

Pilot-scale production of samples was performed at GNT Europa GmbH (Aachen, Germany). Pumpkin colouring concentrate was added at 1.0% *w/w* to the beverage base prepared from an aqueous mixture of invert sugar syrup (7.0% *w/w*), anhydrous citric acid (0.18%), anhydrous trisodium citrate (0.03% *w/w*), and potassium sorbate (antimicrobial) (0.02% *w/w*). To maintain the physical stability against sedimentation, commercial citrus pectin (0.05% *w/w*) was added, prior to mixing for 5 min (Ultra-Turrax, Ystrahl X10/20-750, Ballrechten-Dottingen, Germany). The pH of the beverage was 3.5. The beverage was thermally processed (96 °C, 30 s) (HT220 HTST/UHT, De Meern, The Netherlands) and homogenized (25 MPa) (TwinPanda400, GEA GmbH, Düsseldorf, Germany). Subsequently, the beverage was manually filled into two types of 300 mL clear bottles: monolayer PET bottles and glass bottles (Flaschenland GmbH, Ransbach-Baumbach, Germany). The headspace was minimized as the bottles were filled to the bottle neck. Samples were immediately capped in ambient atmosphere (Bericap SK 38/16 O2S closures, Budenheim, Germany).

### 2.3. Storage of Samples

#### 2.3.1. Dark Storage

Filled sample bottles were stored immediately after processing (time 0) at four isothermal conditions (10 °C and 20 °C for 42 days; 35 °C and 45 °C for 36 days) in temperature-programmable incubators protected from light (Memmert, Germany). Throughout the storage period, seven sampling points were chosen. At each sampling time, one bottle of each type (PET and glass) per storage temperature was taken from the storage, cooled to 4 °C, and analysed immediately for carotenoid content.

#### 2.3.2. Illuminated Storage

The concomitant effect of light and heat (temperature) exposure to carotenoids was tested under accelerated illumination conditions using a temperature-programmable, constant climatic chamber equipped with a UV/Vis light source (KBF-LQC 720, BINDER GmbH, Tuttlingen, Germany) at four temperatures (10 °C, 20 °C, 35 °C, and 45 °C). To be able to study the time-dependent effect, a sample bottle (i.e., one PET and one glass) was taken out of the chamber and the light dosage received was noted down at seven different time points during storage. The samples were exposed to a total light dose of 1.3 megalux-hours (Mlux h), corresponding to 18–19 days of storage inside the chamber. In a real-time scenario, this condition corresponds to exposing the product to a 600-lux light bulb in a 12 h/day-operating supermarket for 180 days (6 months). Seven sampling points were chosen at each sampling point, one bottle of each type (PET and glass) per storage temperature was taken from storage, cooled to 4 °C, and analysed immediately for carotenoid content.

### 2.4. Ascorbic Acid Addition

To confirm whether oxidation was the major cause of carotenoid degradation in the samples, another set of beverage samples was produced, but this time, exogenous ascorbic acid (AA) (300 µg/g beverage) was added to the beverage base prior to processing (cfr. Section 2.2). AA-added samples were packaged in PET bottles and stored at 35 °C in dark and illuminated conditions, as described previously. Only one temperature was evaluated in this case, to study the effect of AA addition to the beverages on carotenoid oxidation.

### 2.5. Carotenoid Extraction and HPLC Analysis

Carotenoids were extracted according to the procedure of Wibowo et al. (2015) with minor modifications. Briefly, 10 mL of sample was mixed with 25 ml extraction solvent (50:25:25 hexane:ethanol:acetone (*v/v/v*) containing 0.1% *w/v* butylated hydroxytoluene (BHT)) and stirred for 20 min. A known concentration of β-Apo-8′-carotenal was added as an internal standard, followed by an addition of 7.5 mL reagent-grade water. After separation into two phases, the upper carotenoid-containing layer was collected and subjected to 1 h of saponification at room temperature with 25 mL of 10% ethanolic potassium hydroxide solution in order to de-esterify the xanthophylls. Afterwards, the extract was washed with 10% sodium chloride solution to remove the alkali. The hexane layer was filtered (Chromafil PET-20/25; Macherey-Nagel, Düren, Germany) and concentrated in a vacuum drying oven (BINDER VD23, Tuttlingen, Germany) at 35 °C for 30 min. The carotenoid residue was redissolved in 400 µL methanol-acetone (2:1 *v/v*). All samples were extracted in duplicate. Carotenoids were separated using a HPLC system (Agilent Technologies 1260 Infinity II) following the same conditions as described by Wibowo et al. (2015). Carotenoid standards (Carotenature^®^, Lupsingen, Switzerland) were used for carotenoid identification (analysing retention times, mass and spectra with commercial standards and literature data) [19,20,21,22] and for quantification (preparation of an eight-point external calibration curve for each carotenoid standard). The method for carotenoid analysis was previously validated (y = 20922–15.51, R^2^ = 0.96). Standard calibration curve correlation coefficients (R^2^) were in the range of 0.9966–0.9999.

### 2.6. Dissolved Oxygen Content Measurement

Dissolved oxygen concentration was monitored during storage using an Optech O_2_ platinum oxygen analyser (MOCON, Minneapolis, MN, USA). For each storage condition, one PET bottle and one glass bottle were equipped with oxygen sensors (attached on the inside of the bottle) before filling. These bottles were exclusively used for the oxygen measurements.

### 2.7. Ascorbic Acid Content Analysis

For samples with added exogenous AA, AA content was analysed exactly as described by Wibowo et al. (2015) using an HPLC-DAD system (Agilent Technologies 1260 Infinity II) equipped with a Prevail C18 column (250 × 4.6 mm, 5 µm particle size) (Grace, Columbia, MD, USA).

### 2.8. Kinetic Modelling

To describe the changes in the main pumpkin carotenoids during storage of the beverage, zero-order (Equation (1)), first-order (Equation (2)), and fractional conversion (Equation (3)) models were used. To identify the appropriate model, a visual inspection of the model fit and residual plot was performed, combined with the estimation of the coefficients of determination (r^2^_adj_) and standard error of estimate (SEE). The temperature dependence of the reaction rate constants was quantified by the activation energy ($E_{a}$) and expressed by the Arrhenius equation (Equation (4)). All kinetic parameters were estimated by one-step nonlinear regression analysis using SAS software (SAS v9.4, 2013, SAS Institute, Cary, NC, USA).

(1) $$C_{t}=C_{0}-k_{T}t$$

(2) $$C_{t}={\;C}_{0}\;\exp\left(-k_{T}t\right)$$

(3) $$C_{t}={\;C}_{\infty }+(C_{0}-C_{\infty })\;\exp\left(-k_{T}t\right)$$

(4) $$k_{T}={\;k}_{\mathrm{Tref}}\exp\left[\frac{E_{a}}{R}\;\left(\frac{1}{T_{\mathrm{ref}}}-\frac{1}{T}\right)\right]$$

In these equations, $C_{t}$ represents the carotenoid concentration (µg/g beverage) at a time t, $C_{0}$ is the initial carotenoid concentration at t = 0 (i.e., at the start of the storage experiment), $C_{\infty }$ is the final carotenoid concentration at extended storage time, $k_{T}$ is the apparent reaction rate constant at a certain temperature T, $k_{\mathrm{Tref}}$ is the rate constant at the reference temperature (25 °C), and $E_{a}$, T, and R denote the activation energy (kJ/mol), temperature (K), and gas constant (8.314 J·mol^−1^·K^−1^), respectively.

To establish the kinetic models for carotenoid concentration changes under light exposition, changes in experimental concentrations of each all-trans-carotenoid were first modelled as a function of light dosage (0 to 1.3 Megalux-hour, Mlux h) received by the sample at each sampling moment. The light dosage accumulation in the climatic chamber considers lamp aging during the tests and allows compensation for the effects of both reduction in light intensity over time and temperature on light intensity. Hence, the number of storage days required to achieve a specific light dosage inside the chamber varied from one storage temperature to another, so light dosage data were used to establish the kinetic models rather than actual storage days. For this, the parameter t in Equations (1)–(3) was expressed in terms of light dosage. Secondly, the values obtained in the previous step were used to generate predictive models for the changes in carotenoid concentrations as a function of storage days, taking into account the real-time equivalent scenario (i.e., 600 Lux light bulb in a supermarket operating for 12 h/day) of the conditions simulated inside the climatic chamber as described previously.

To evaluate the statistical confidence and correlation between the jointly estimated parameters ($k_{\mathrm{Tref}}$ and $E_{a}$) for each trans-carotenoid, 90% joint confidence regions (JCR) were constructed (Equation (5)).

(5) $$\mathrm{SSQ}\;\leq \mathrm{SSQ}\left(\theta \right)\left\{1+\frac{p}{m-p}\;F\;\left(p,\;m-p,\;1-\varphi \right)\right\}$$

where $\mathrm{SSQ}\left(\theta \right)$ represents the error sum of squares associated with least-squares estimate θ, p the number of parameters estimated simultaneously, m the number of observations, and F the upper 1−φ quantile for an F distribution with p and m−p degrees of freedom [23].

## 3. Results and Discussion

Despite being lipophilic, carotenoids frequently occur in highly aqueous systems like fruit or vegetable juices. Therefore, an aqueous dispersion closely simulates a food product rather than systems in which carotenoids were dissolved in oil or in an organic solvent. To mimic an aqueous dispersion, a still beverage was manufactured with the addition of pumpkin juice concentrate as a colourant. The choice of a pumpkin colouring concentrate permitted the simultaneous investigation of heat and light stability of several trans-carotenoids (i.e., all-trans-beta-carotene, all-trans-antheraxanthin, all-trans-lutein, all-trans-violaxanthin and all-trans-neoxanthin). An intense and stable yellow-orange colour is an important requirement for the attractiveness of such products over their shelf-life. This quality attribute is strictly related to the fate of the carotenoids during storage. Model beverages stored at 20 °C in both dark and illuminated conditions are shown in Figure S1 (Supplementary Material).

### 3.1. Changes in Carotenoids during Dark Storage

#### 3.1.1. Changes in All-Trans-Carotenoid Concentrations

**Figure 1:**
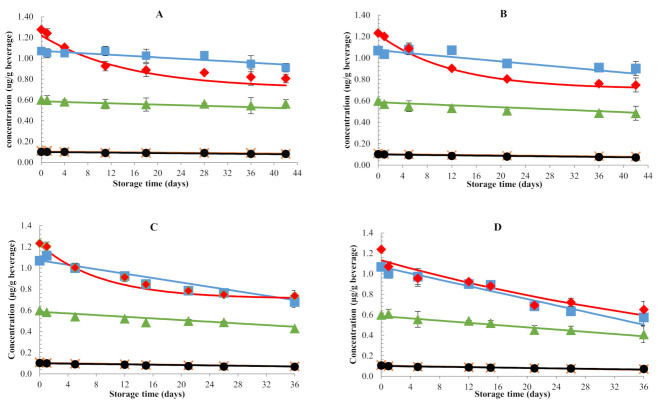
Changes in all-trans-carotenoid concentration of samples packaged in glass bottles during storage in darkness at: (**A**) 10 °C; (**B**) 20 °C; (**C**) 35 °C; and (**D**) 45 °C. ♦ trans-beta-carotene; ■ trans-antheraxanthin; ▲ trans-lutein; ● trans-violaxanthin; ✖ trans-neoxanthin.

Figure 1 shows the changes in carotenoid concentrations in samples packaged in glass bottles during storage in darkness at different temperatures. Concentrations of all-trans-carotenoids showed declining trends as a function of time at rates depending on storage temperature. Degradation already took place at the lowest temperature studied (10 °C) but became more pronounced as the storage temperature increased. For example, a final loss of 30–32% from the initial all-trans-β-carotene content was observed after storage at 10 °C and 20 °C for 42 days, while 36–38% was lost after 36 days of storage at 35 °C and 45 °C. Final losses of all-trans-antheraxanthin were smaller, being around 14%, 19%, 30%, and 38% of the initial content after storage at 10 °C, 20 °C, 35 °C and 45 °C, respectively, for 36–42 days. This is in line with previous studies in which carotenoid losses were reported upon storage in darkness at refrigerated, ambient, and high temperatures. For instance, Lin et al. (2005) reported all-trans-β-carotene losses of approximately 78, 81 and 95% in canned tomato juice stored at 4, 25, and 35 °C for 3 months, while Odriozola-Serranno et al. (2008) [24] reported 70% of losses of total carotenoids in tomato juices packed in polypropylene bottles stored at 4 °C for 3 months. In commercial fruit beverages, a 25% decrease of total carotenoids was observed for juices stored in darkness at 4 °C for 12 days [25], while 9–21% of losses of total carotenoid contents in orange juice during refrigerated storage for 10 days was reported [26]. In contrast to our results, Wibowo et al. (2015) and García–Alonso et al. (2009) reported very limited carotenoid losses in pasteurized orange juice and commercially available tomato juice, respectively, during dark storage at 20–37 °C. This was mainly attributed to ascorbic acid naturally present in these foods which was able to protect the carotenoids from oxidation.

Kinetic modelling was performed to describe the concentration changes in all-trans-carotenoids as a function of time during dark storage at four temperatures (Figure 1). As expected, faster carotenoid losses were observed at higher temperatures, represented by higher degradation rate constants (k-values) (Table 1). This observation is in agreement with other studies [9,10,27,28,29]. At the beginning of storage, all-trans-β-carotene was the most sensitive carotenoid, showing higher losses compared to the others. However, after approximately 28 days of storage at each temperature studied, the concentration changes declined at a slower rate. Hence, the experimental data were more adequately fitted by a first-order fractional conversion model, although the plateau value was not yet reached in some cases due to the short duration of storage (36–42 days). Based on literature, it has been noted that β-carotene degradation follows different kinetic models depending on, for example, β-carotene type, substrate compound, oxygen exposure, or heat treatment [13]. The presence of a plateau value at prolonged storage times is most probably due to the antioxidant activity of β-carotene, which could be able to protect itself from oxidation for a certain time by acting both as a physical quencher and a radical trapping. Meanwhile, changes in all-trans-antheraxanthin, all-trans-lutein, all-trans-violaxanthin, and all-trans-lutein were fitted to zero-order models. Temperature dependence of the rate constants (k) could be adequately described by an Arrhenius relationship (r^2^_adj_ = 0.81–0.98). Activation energies were for all carotenoids lower than 50 kJ/mol, which, according to Manzocco et al. (2012) [30], indicates the temperature to be a scarcely accelerating factor.

When analysing the degradation rate constants of each carotenoid modelled by a zero-order equation, results suggest that all-trans-antheraxanthin degraded the fastest (highest k) and its k-value showed greater temperature sensitivity (highest E_a_). To statistically compare the simultaneously estimated parameters (k_Tref_ and E_a_) of these four carotenoids, 90% joint confidence regions (JCR) were constructed (Figure S2, Supplementary Material). The JCR (α = 0.1) of all-trans-antheraxanthin confirmed the previous observation since there was no overlap of its JCR with the other carotenoids (Figure S2A,B). Based on the estimated k-values, all-trans-lutein showed around a 2-fold times higher stability than all-trans-antheraxanthin, which was in agreement with the results of Hadjal et al. (2013) and Aparicio–Ruiz et al. (2011, 2012) in blood orange and oil model juice systems, respectively. The JCRs of all-trans-violaxanthin and all-trans-neoxanthin overlapped, suggesting no significant differences in their k_Tref_ and E_a_-values. Both had significantly lower k-values compared to all-trans-antheraxanthin and all-trans-lutein, suggesting higher stability during dark storage. This observation might be attributed to violaxanthin and neoxanthin being highly esterified in the pumpkin juice concentrate. Hadjal et al. (2013) noted that the esterification of blood orange xanthophylls in aqueous model systems gives them higher stability against degradation. Aparicio–Ruiz et al. (2011, 2012) reported that among these epoxycarotenoids (i.e., antheraxanthin, violaxanthin and neoxanthin) dissolved in olive oil, all-trans-neoxanthin exhibited the greatest thermal degradation stability, which was in agreement with our results. However, the fact that all-trans-violaxanthin and all-trans-neoxanthin concentrations were much lower compared to all-trans-antheraxanthin and all-trans-lutein, added also, to some extent, uncertainties in analysing changes in these two carotenoids.

#### 3.1.2. Changes in Isomer Concentrations

Particular isomer chromatographic peaks were tentatively identified by analysis of spectral characteristics and comparison with literature data [22,27,31].

**Figure 2:**
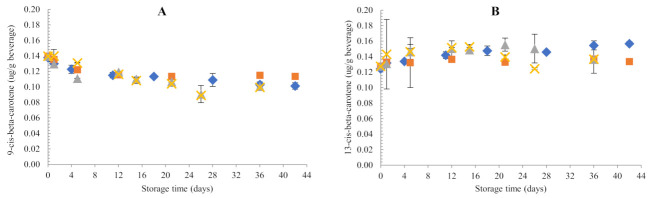

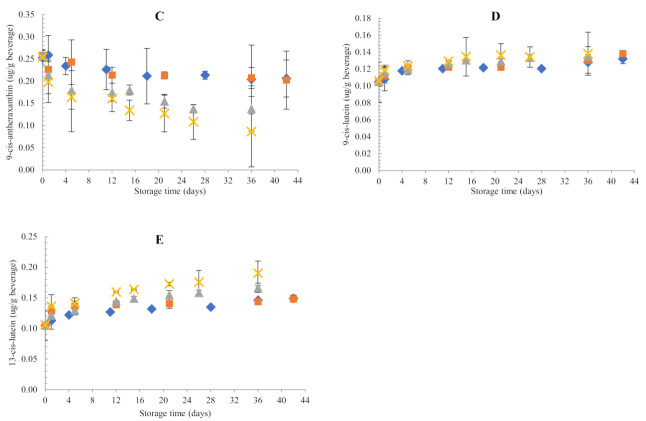
Changes in cis-carotenoid concentrations of samples packed in glass bottles during storage in darkness. (**A**) 9-cis-β-carotene; (**B**) 13-cis-β-carotene; (**C**) 9-cis-antheraxanthin; (**D**) 9-cis-lutein; (**E**) 13-cis-lutein. Storage temperature: ♦ 10 °C; ■ 20 °C; ▲ 35 °C; ✖ 45 °C.

Isomer peaks were initially present in the samples, possibly due to the inherent content in the pumpkin juice concentrate and/or due to processing. Throughout storage, no formation of new isomers nor epoxide derivatives from all-trans-carotenoid degradation were observed, suggesting that storage in darkness at the temperatures studied did not initiate new formation pathways of isomers. Small, but non-significant changes in the concentrations of isomers initially present in the sample were observed (Figure 2). Concentrations of 9-cis-antheraxanthin decreased throughout storage, and was more pronounced at 45 °C (from initial 0.26 µg/g to a final concentration of 0.10 µg/g). Meanwhile, 9-cis-β-carotene and 13-cis-β-carotene concentrations showed less pronounced changes, with values ranging from 0.10 to 0.15 µg/g throughout the whole storage period. Similarly, no clear trend was observed for 9-cis-lutein and 13-cis-lutein, whose concentrations ranged from 0.10–0.17 µg/g beverage during storage. Concentrations of cis-violaxanthin and cis-neoxanthin were not quantified as they were below the quantification limits. Since all these isomers were initially present prior to storage, the concentrations quantified for each cis-isomer was attributed to the simultaneous formation and degradation of the isomer itself or the corresponding all-trans-carotenoid. Hence, the isomer concentration coming solely from all-trans-degradation, cis-isomer formation, or cis-isomer degradation could not be determined. This complex behaviour of isomerisation and degradation was reported before by other researchers who studied carotenoid changes in fruit juices during dark storage at 4–35 °C [32,33,34]. Besides isomerisation and degradation, disappearance of all-trans- or cis-carotenoids can also be explained by the formation of volatile and low molecular weight compounds not detected by HPLD-DAD, as observed by Zepka et al. (2009) in the cashew apple juice model system and Limbo et al. (2007) for the aqueous β-carotene system.

Since this study did not quantify specific degradation products of carotenoid oxidation, it is not possible to further unravel the reaction mechanism. Based on the reaction mechanisms deduced from literature, it is supposed that isomerisation could be the first step of oxidation, leading to a diradical of the carotenoid which can easily be attacked by oxygen on either side of the cis-bond. This radical attack, followed by homolytic internal substitution, yields epoxides from which the final products (e.g., apocarotenones and apocarotenals) could be formed [6].

### 3.2. Changes in Carotenoids during Illuminated Storage

#### 3.2.1. Changes in All-Trans-Carotenoid Concentrations

Under high-intensity illumination (1875–3000 lux), colour fading of the beverage was observed in a shorter time period compared to the beverage stored in dark (Figure S1, Supplementary material). This demonstrated that light accelerates colour depletion of photosensitive foods, such as carotenoid-containing beverages, affecting their quality. Consequently, the effect of light on quality losses in carotenoid-containing products during storage cannot be underestimated and should be considered when performing shelf-life studies.

**Figure 3:**
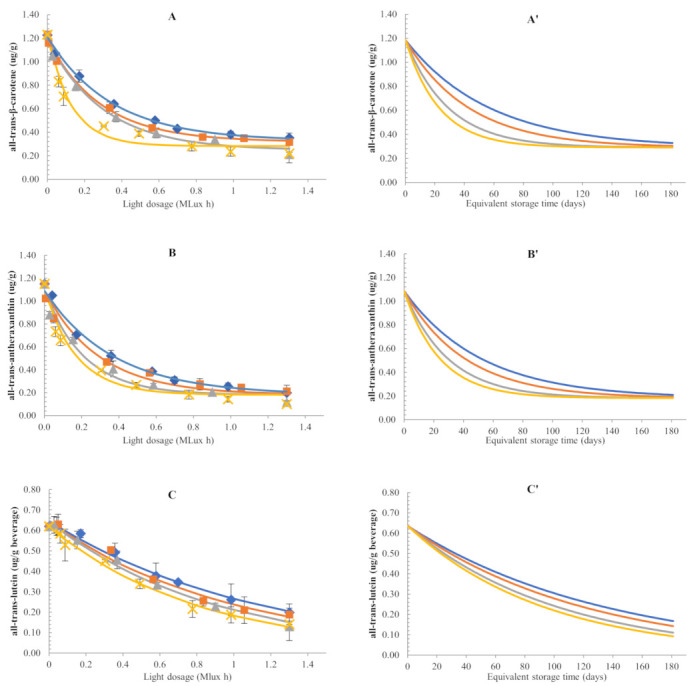

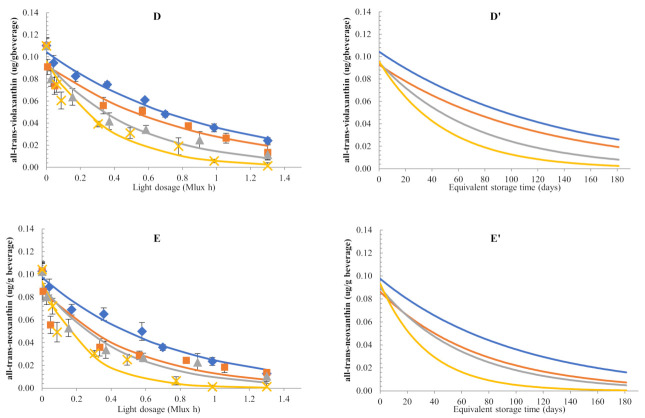
Changes in carotenoid concentrations of a pumpkin-based beverage packed in glass bottles during storage at illuminated condition as a function of light dosage: (**A**) all-trans-β-carotene; (**B**) all-trans-antheraxanthin; (**C**) all-trans-lutein; (**D**) all-trans-violaxanthin; and (**E**) all-trans-neoxanthin. Letters with apostrophe (**A′**, **B′**, **C′**, **D′**, and **E′**) show the predictive modelling of the changes of each corresponding all-trans-carotenoid as a function of equivalent storage time (days), assuming that the beverage is displayed in a retail shelf lit with a 600-lux bulb for 12 h/day. Storage temperatures: ♦ and 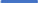 10 °C; ■ and 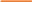 20 °C; ▲ and 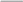 35 °C; ✖ and 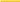 45 °C.

The expected instability of carotenoids as a function of light dosage (Mlux h) received by the sample during illuminated storage was well-demonstrated (Figure 3). Changes in concentrations of all-trans-β-carotene and all-trans-antheraxanthin fitted best to the fractional conversion model while changes in trans-lutein, trans-violaxanthin and trans-neoxanthin were modelled using a first-order equation. For each trans-carotenoid, k-values (Mlux h^−1^) increased with storage temperature (Table 2), leading to progressive colour fading. Temperature-dependency of the k-values was well-described by the Arrhenius equation during illuminated storage (R^2^ of 0.96–0.99), suggesting that the accelerating effect of light on carotenoid degradation rates was affected by temperature. Similar to E_a_ values obtained for dark storage, E_a_ values for carotenoid degradation under light exposure were also lower than 50 kJ/mol indicating a relatively small accelerating effect of carotenoid degradation at elevated storage temperature.

Based on JCR analysis (Figure S3, Supplementary material), k_Tref_ and E_a_ combinations are not significantly different for all-trans-β-carotene and all-trans-antheraxanthin, suggesting similar stability under the studied conditions. Meanwhile, all-trans-lutein, with significantly lower k and E_a_, appeared to be a more stable carotenoid than all-trans-violaxanthin and all-trans-neoxanthin under the given conditions. Similarly, Xiao et al. (2018) reported significantly higher stability of all-trans-lutein compared to all-trans-β-carotene for carotenoid standards dissolved in hexane and exposed to light (1800 lux) at 25–45 °C. Chemical stability of carotenoids has been reported to be partly influenced by their structure [35]. Generally, the decreasing number of coplanar conjugated double bonds and the presence of hydroxy and keto groups decrease carotenoid reactivity in radical-scavenging reactions. Therefore, β-carotene is generally expected to be more stable than hydroxycarotenoids (e.g., lutein) due to the absence of hydroxyl groups (Table S1). However, this expected influence of structure on carotenoid stability could not be established in the present study. The kinetic parameters of all-trans-β-carotene and all-trans-lutein could not directly be compared due to the different kinetic models used. Regarding the kinetic aspect of the epoxycarotenoid degradation (antheraxanthin, violaxanthin and neoxanthin) few studies are available comparing their stability with the one of β-carotene. In those studies, k and E_a_-values were shown to be very matrix-dependent [36,37]. Nevertheless, our results are in agreement with a number of literature studies demonstrating the catalytic effect of light on carotenoid degradation. For instance, Limbo et al. (2007) reported that light was a critical variable in the degradation of β-carotene in an aqueous medium at 33 °C even in the presence of very low oxygen partial pressure inside the packaging. Moreover, they also correlated carotenoid degradation to illuminance and UVA irradiance values of three different lamps, suggesting that carotenoid degradation occurred faster for samples exposed to light sources with higher energy emission on the blue region (450–485 nm) of the spectrum. This was also the case in our experimental set-up, in which the light bulbs (Osram Biolux T8 30W/965) inside the climatic chamber emitted the highest illuminance at 440–450 nm.

So far, the present results showed that the kinetic parameters regarding for carotenoid changes in a beverage stored under dark conditions cannot be used to predict the behaviour of the same beverage exposed to light. However, from an industrial point of view, the results obtained from this study would be of more practical importance if the information on light dosage could be interpreted in terms of storage days in a supermarket shelf, which will be discussed in Section 3.3.

#### 3.2.2. Changes in All-Trans-Carotenoid Concentrations

**Figure 4:**
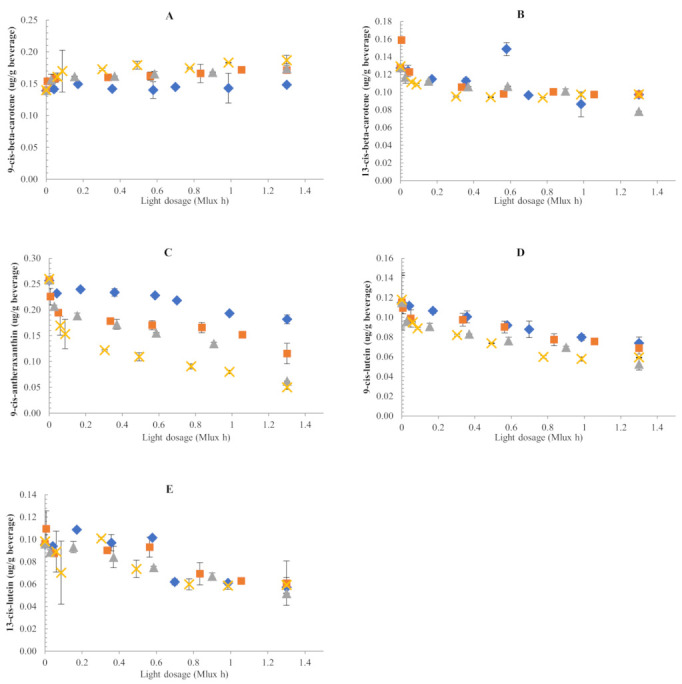
Changes in cis-carotenoid concentrations of samples packed in glass bottles during illuminated storage. (**A**) 9-cis-β-carotene; (**B**) 13-cis-β-carotene; (**C**) 9-cis-antheraxanthin; (**D**) 9-cis-lutein; (**E**) 13-cis-lutein. Storage temperature: ♦ 10 °C; ■ 20 °C; ▲ 35 °C; ✖ 45 °C.

Similar to dark storage, isomeric profiles were not altered during illuminated storage and no new isomer peaks were observed at the temperature range studied. Moreover, based on the experimental concentrations measured, the loss of total all-trans-carotenoids was not compensated by the other isomers formed. Experimental cis-isomer concentrations showed small and inconsistent changes during storage under illuminated conditions (Figure 4). Kinetic modelling was not performed on the data obtained.

A declining trend was observed for 9-cis-antheraxanthin whose concentration changed from initially 0.26 µg/g to 0.05 µg/g at the end of illuminated storage at 45 °C. Meanwhile, 13-cis-β-carotene and 13-cis-lutein did not exhibit significant variations. A slight accumulation of 9-cis-β-carotene was observed, increasing initially from 0.13 µg/g to 0.20 µg/g at 45 °C. Similarly, an increase of 9-cis-β-carotene was reported for tomato juice stored under illuminated condition (2500 lux) at 4, 25, or 35 °C [32]. Pesek et al. (1987) [38] observed no new isomer peak formation during photodegradation of carotenoids in a model vegetable juice system after 8 days of intense light exposure (2475 lux light intensity) at 4 °C. They assumed that if isomerisation was occurring, either cis-isomers were co-eluting with the trans-peaks or were present at levels below the detection limit. Nonetheless, the present results demonstrated that carotenoid degradation was mainly driven by oxidation under the experimental conditions used.

### 3.3. Predictive Modelling of Carotenoid Degradation in Both and Illuminated Conditions

In order to translate our previous results to real-life scenarios wherein the beverage is displayed in highly lit shelves (600–800 lux) to attract consumers, the kinetic parameters obtained from Section 3.2.1 were used to derive another set of models and parameters which allowed the expression of concentration changes a function of storage days (Figure 3A′–E′). This step allowed us to study the acceleration induced by light exposition with respect to dark storage and allowed one to quickly extrapolate the carotenoid losses to actual storage conditions in the supermarket shelves.

**Figure 5:**
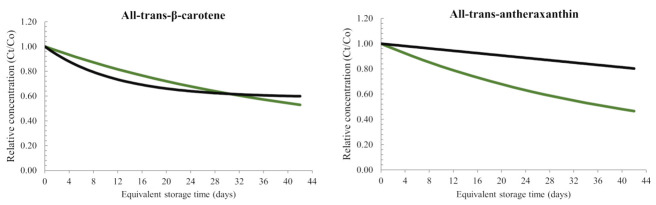

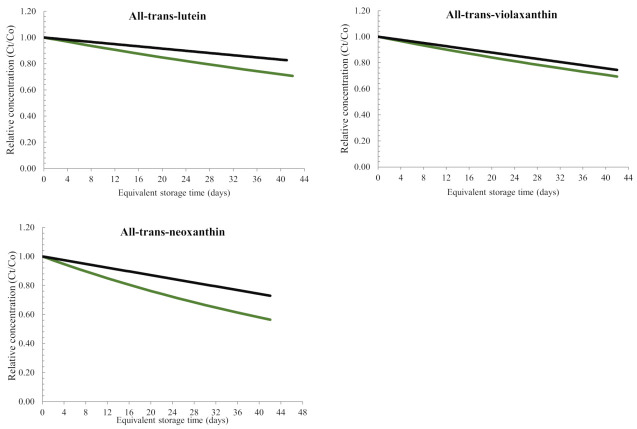
Predictive models of carotenoid concentration changes of samples packed in glass bottles during storage at dark (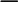) and illuminated (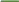) conditions as a function of equivalent storage time (days) at 20 °C. Carotenoid concentrations (µg/g beverage) were expressed in relative terms ($C_{t}/C_{0}$) where $C_{t}$ represents the carotenoid concentration at a time t (equivalent storage days), $C_{0}$ is the initial carotenoid concentration at t = 0 (i.e., at the start of the storage experiment, day 0).

The predictive models for all-trans-carotenoid changes during dark and illuminated conditions at 20 °C are illustrated in Figure 5. Analogously to what was previously observed and based on the predictive kinetic parameters, light induced higher rates of carotenoid losses compared to dark storage within the same timeframe. This can be explained by the different mechanisms involved in carotenoid oxidation. In dark, carotenoid oxidation follows a radical pathway (autoxidation) that can be induced by temperature and radical starters (e.g., metals or minute amounts of contaminants in the water or in other ingredients). In illuminated conditions, two parallel reactions are reported to occur: a free radical autoxidation and a photooxidation. In the latter pathway, carotenoids are converted to the triplet excited state upon absorption of high-energy light, allowing them to react with triplet molecular oxygen to form a highly reactive singlet oxygen [13]. Hence, although the observation was limited by the short duration of dark storage, the results clearly demonstrated the accelerating effect of light on carotenoid degradation. To the best of our knowledge, this is the first study in which kinetic models for carotenoid concentration changes were expressed as a function of light dosage, and predictive models as a function of equivalent storage days were generated thereafter.

### 3.4. Impact of Packaging and Dissolved Oxygen Content in the Samples on the Changes in All-Trans-Carotenoid Concentrations

Interestingly, for storage in both dark and illuminated conditions, the type of packaging material did not have a significant impact on the rates of carotenoid losses. The k_Tref_ and E_a_ values for each all-trans-carotenoid degradation are generally slightly higher, albeit not significant (α = 0.1), for samples packaged in glass compared to the ones in PET bottles (Figures S2 and S3 graphs C–F, Supplementary material). As the kinetic parameters were of the same order of magnitude between the two types of bottles, our results suggest that oxygen diffusion through the packaging material was not a rate-limiting factor for the carotenoid losses observed in our study. This observation is probably due to the high initial amount of oxygen present within the sample after processing and packaging. Initially, the samples had a dissolved oxygen content ranging from 6.5–7.3 mg/L, corresponding to a beverage saturated with oxygen. According to Vidal et al. (2006) [39], oxygen exchange kinetics within a bottle (headspace and dissolved oxygen) outweighs the oxygen diffusion kinetics from the external atmosphere as long as the total oxygen inside the bottle has not been completely used by oxygen-consuming reactions in the sample.

Given that carotenoid degradation is greatly affected by oxygen [6], dissolved oxygen content throughout storage was monitored in the samples as a function of time and light dosage during dark and illuminated conditions (data not shown). For all temperatures studied, dissolved oxygen content decreased as a function of storage time or light dosage, the decline in dissolved oxygen being slower at lower temperatures. A high correlation was obtained between dissolved oxygen content and all-trans-carotenoid losses (*p* < 0.0001, r^2^_adj_ = 0.93–0.97) in both dark and illuminated storage. Thus, the decrease in dissolved oxygen content in the samples could be attributed to its consumption via carotenoid oxidation. Some studies reported that, even at a very low oxygen content inside the packaging, carotenoid oxidation still occurred [13,40]. For instance, Vásquez–Caicedo et al. (2007) reported that dissolved residual oxygen was responsible for carotenoid degradation during 98-day dark storage of hot-filled and nitrogen-flushed, canned mango purees at 25–30 °C. The function of equivalent storage days was generated thereafter.

### 3.5. Impact of Ascorbic Acid Addition to Changes in All-Trans-Carotenoid Concentration

In order to prove that carotenoid losses in the samples could be greatly attributed to oxidation, a follow-up experiment was set up, wherein a water-soluble antioxidant (i.e., ascorbic acid, AA) was added to the beverage prior to processing. In the presence of oxygen, AA undergoes aerobic degradation, thereby diminishing the available oxygen to initiate carotenoid oxidation.

Upon addition of exogenous AA to the juice samples prior to processing, individual carotenoid content gradually decreased as a function of storage time and light dosage for dark and illuminated conditions, respectively. Compared to the k-values of non-AA samples, the k-values calculated for AA-added samples were about two- to four-fold lower in dark storage, while it was two to seven times lower for illuminated storage (Table 3). These observations were related to the protective action of AA on carotenoid degradation, as documented in previous studies [15,16,26,41,42,43,44].

Changes in AA concentrations and dissolved oxygen content of AA-added samples during storage were monitored. Initially, AA was added to the beverage base at a 300 µg/g beverage (300 ppm). After processing and packaging, AA content in the sample decreased by approximately 48%, which can be ascribed to the enhanced AA oxidation at high processing temperatures [17]. Indeed, there was an initial fast and large decrease of dissolved oxygen right after processing (decrease from 7.3 mg/L to 1.6 mg/L), which coincided with the drastic decrease in AA concentration. During storage, AA content in the samples decreased gradually as a result of its oxidative degradation. Plots of AA concentration as a function of storage time or light dosage during dark or illuminated storage condition, respectively, best fitted a first-order model (data not shown). Moreover, multiple regression analysis of the data indicated that the AA content significantly correlated (*p* < 0.001, r^2^ = 0.78) to the level of dissolved oxygen. Similar findings have been reported by Solomon et al. (1995) [45] the changes in ascorbic acid in orange juice stored at 8 °C.

Additional calculations (mole: mole ratios) were done to evaluate the correlation between AA degradation and oxygen consumption in the AA-added samples, considering that 0.5 mole of oxygen (O_2_) is needed for the aerobic oxidation of one mole of AA. The results (data not shown) indicated that, right after processing and during the first 5 days of dark storage, approximately 100% of the consumed dissolved oxygen was used by AA oxidation, assuming that aerobic oxidation was the only degradation pathway at the beginning of storage. This indicates that AA addition diminished the available oxygen needed for other oxygen-consuming reactions in the samples, which was mainly carotenoid oxidation. In a more cognitive perspective, further research is needed to understand if there are other chemical interactions between AA and carotenoids that could explain the stabilizing effect of the former to the latter [9]. For industrial applications, the use of AA to stabilize carotenoids in a product can be an important consideration.

## 4. Conclusions

This study aimed to investigate the degradation kinetics of the main carotenoids in a pumpkin-based colouring concentrate. For this, a still beverage product coloured with pumpkin juice concentrate was used. It had the advantage of simplicity for testing the impacts of light (dark vs. illuminated) and temperature (10, 20, 35 and 45 °C) on carotenoid losses in a photosensitive food product during storage. This information is fundamental in predicting carotenoid losses and its relation to shelf-life of the beverage product during storage especially at retail conditions. Uni-response modelling of the experimental data allowed the estimation of kinetic parameters (k and E_a_) expressing the changes in all-trans-carotenoids at different storage conditions. Generally, carotenoid losses in the product were influenced by both storage temperature and light exposure. Besides, given the relatively limited changes in isomer concentrations, this study showed that the degradation kinetics was driven by oxidative reactions. Changes in the dissolved oxygen content were highly correlated with all-trans-carotenoid losses for all storage conditions. It was demonstrated that the addition of ascorbic acid, which degrades via an oxygen-consuming pathway, had a protective effect on the carotenoids in the beverage. For industrial applications, modified atmosphere techniques, the use of antioxidants (water- or lipid-soluble), as well as the choice of light bulbs in retail shelves, need to be considered to reduce carotenoid losses in photosensitive food products.

## Figures and Tables

**Table 1:** Estimated kinetic parameters describing the changes in all-trans-carotenoid concentrations during storage in dark conditions in two kinds of packaging materials (glass versus PET bottles). T_ref_ = 25 °C.

Carotenoid	Temp. (°C)	Glass					PET				
		C_0_ (µg/g)	k(×10^−2^) ^a^	k_ref_ (×10^−2^) ^a^	E_a_ (kJ/mol)	r^2^_adj_	C_O_ (µg/g)	k(×10^−2^) ^a^	k_ref_ (×10^−2^) ^a^	E_a_ (kJ/mol)	r^2^_adj_
All-trans-β-carotene Fractional conversion	10	1.22 ± 0.01	5.41	7.50 ± 1.10	15.13 ± 3.44	0.98	1.15 ± 0.03	5.19	8.40 ± 2.0	22.55 ± 6.91	0.96
	20		6.73					7.19			
	35		9.11					11.29			
	45		10.97					14.89			
All-trans-antheraxanthin Zero order	10	1.07 ± 0.01	0.30	0.64 ± 0.06	35.48 ± 3.53	0.93	1.04 ± 0.03	0.37	0.78 ± 0.13	35.23 ± 6.50	0.80
	20		0.50					0.61			
	35		1.02					1.23			
	45		1.60					1.90			
All-trans-lutein Zero order	10	0.59 ± 0.01	0.16	0.28 ± 0.01	26.75 ± 3.74	0.87	0.59 ± 0.01	0.13	0.31 ± 0.08	41.16 ± 9.67	0.71
	20		0.23					0.25			
	35		0.39					0.52			
	45		0.55					0.87			
All-trans-violaxanthin Zero order	10	0.10 ± 0.01	0.05	0.07 ± 0.01	19.48 ± 3.81	0.81	0.10 ± 0.01	0.07	0.11 ± 0.01	22.85 ± 4.08	0.81
	20		0.06					0.09			
	35		0.09					0.14			
	45		0.12					0.19			
All-trans-neoxanthin Zero order	10	0.10 ± 0.01	0.05	0.07 ± 0.01	12.88 ± 3.31	0.82	0.10 ± 0.01	0.06	0.10 ± 0.01	26.20 ± 3.95	0.85
	20		0.06					0.08			
	35		0.08					0.14			
	45		0.10					0.19			

^a^ Unit of k = day^−1^ for fractional conversion model and µg/g·day^−1^ for zero order model. k-values at different temperatures were calculated by one-step nonlinear regression analysis.

**Table 2:** Estimated kinetic parameters describing the changes in all-trans-carotenoid concentrations during storage in illuminated conditions in two kinds of packaging materials (glass versus PET bottles). T_ref_ = 25 °C.

Carotenoid	Temp. (°C)	Glass					PET				
		C_0_ (µg/g)	k(×10^−2^) ^a^	k_ref_ (×10^−2^) ^a^	E_a_ (kJ/mol)	r^2^_adj_	C_O_ (µg/g)	k(×10^−2^) ^a^	k_ref_ (×10^−2^) ^a^	E_a_ (kJ/mol)	r^2^_adj_
All-trans-β-carotene Fractional conversion	10	1.19 ± 0.02	2.44	3.68 ± 0.29	19.25 ± 2.58	0.96	1.16 ± 0.02	2.54	3.69 ± 0.35	17.43 ± 3.14	0.96
	20		3.22					3.27			
	35		4.73					4.63			
	45		6.00					5.73			
All-trans-antheraxanthin Fractional conversion	10	1.08 ± 0.02	2.68	3.79 ± 0.33	16.20 ± 2.87	0.98	1.06 ± 0.03	2.75	3.51 ± 0.44	11.50 ± 3.94	0.96
	20		3.39					3.25			
	35		4.68					4.08			
	45		5.71					4.70			
All-trans-lutein First order	10	0.64 ± 0.01	1.02	1.21 ± 0.03	7.86 ± 1.21	0.99	0.67 ± 0.01	1.24	1.50 ± 0.04	8.36 ± 1.52	0.99
	20		1.15					1.40			
	35		1.35					1.66			
	45		1.48					1.84			
All-trans-violaxanthin First order	10	0.10 ± 0.01	1.52	2.49 ± 0.11	22.94 ± 3.58	0.96	0.10 ± 0.01	1.79	3.25 ± 0.10	27.90 ± 3.96	0.98
	20		2.12					2.68			
	35		3.35					4.68			
	45		4.44					6.60			
All-trans-neoxanthin First order	10	0.09 ± 0.01	2.08	3.29 ± 0.17	21.44 ± 3.97	0.97	0.09 ± 0.01	2.12	3.43 ± 0.17	22.42 ± 3.91	0.98
	20		2.84					2.94			
	35		4.36					4.60			
	45		5.67					6.06			

^a^ Unit of k = Mlux h^−1^ for fractional conversion model and µg/g·day^−1^ for zero order model. k-values at different temperatures were calculated by one-step nonlinear regression analysis.

**Table 3:** Comparison of degradation rate constants (k) at 35 °C of all-trans-carotenoids in samples without and with added ascorbic acid (AA) during dark and illuminated storage.

Carotenoid	Kinetic Model Order (Dark)	k (×10^−2^) * (Dark Storage)		Kinetic Model Order (Illuminated)	k * (Illuminated Storage)	
		Without Added AA	With Added AA		Without Added AA	With Added AA
All-trans-β-carotene	First-order	1.70 ± 0.20 ^a^	0.39 ± 0.01 ^b^	First-order	1.68 ± 0.10 ^a^	0.23 ± 0.03 ^b^
All-trans-antheraxanthin	Zero-order	1.23 ± 0.06 ^a^	0.79 ± 0.11 ^b^	First-order	2.31 ± 0.10 ^a^	0.28 ± 0.05 ^b^
All-trans-lutein	Zero-order	0.52 ± 0.01 ^a^	0.28 ± 0.08 ^b^	First-order	1.34 ± 0.06 ^a^	0.26 ± 0.03 ^b^
All-trans-violaxanthin	Zero-order	0.14 ± 0.01 ^a^	0.09 ± 0.01 ^b^	First-order	2.06 ± 0.11 ^a^	0.78 ± 0.05 ^b^
All-trans-neoxanthin	Zero-order	0.14 ± 0.01 ^a^	0.08 ± 0.01 ^b^	First-order	2.75 ± 0.17 ^a^	1.30 ± 0.12 ^b^

* Units of k: during dark storage, k = day^−1^ for fractional conversion model and µg/g·day^−1^ for zero order equation; during illuminated storage, k = Mlux h^−1^ for first order equation. Significant difference between the two mean k values (without added AA vs. with AA) for each storage condition (dark and illuminated) storage are indicated by letters ^a^ and ^b^ (*t*-test at significance *p* ≤ 0.05 level).

## Data Availability

The data used for the figures are available on 10.5281/zenodo.5986981 accessed on 3 February 2022.

## Supplementary Materials

The following supporting information can be downloaded at: https://www.mdpi.com/article/10.3390/foods11030485/s1, Figure S1: Model beverage samples during storage at 20 °C in both (**A**) dark and (**B**) illuminated conditions; Figure S2: 90% Joint confidence regions of the estimated kinetic parameters (k_ref_ and E_a_) for the changes in all-trans-carotenoid which were modelled using the same kinetic model during dark storage in (**A**) glass bottles and (**B**) PET bottles; Figure S3: 90% Joint confidence regions (JCR) of the estimated kinetic parameters (k_ref_ and E_a_) for the changes in all-trans-carotenoid which fitted to similar kinetic order models during illuminated storage in glass bottles. Table S1. Chemical structure and absorption maxima of all-trans-carotenoids studied in the pumpkin juice concentrate-coloured beverage.
