# Supplementary material for: Heat and Light Stability of Pumpkin-Based Carotenoids in a Photosensitive Food: A Carotenoid-Coloured Beverage
Source: Foods. 2022 Feb 7;11(3):485. doi: 10.3390/foods11030485 (PMC8834637; doi:10.3390/foods11030485)
Supplement: Supplementary file 1 [file foods-11-00485-s001.zip › foods-1580531-supplementary.pdf]

Supplementary Materials

Heat And Light Stability of Pumpkin-Based Carotenoids in a Photosensitive Food: A Carotenoid-Coloured Beverage

Sharmaine Atencio[a*], Sarah H.E. Verkempinck[a], Kai Reineke[b], Marc Hendrickx[a], Ann Van Loey[a]

[a] KU Leuven, Department of Microbial and Molecular Systems, Laboratory of Food Technology, Kasteelpark Arenberg 22, Box 2457, 3001, Leuven, Belgium

[b] GNT Europa GmbH, Kackertstrasse 22, 52072 Aachen, Germany

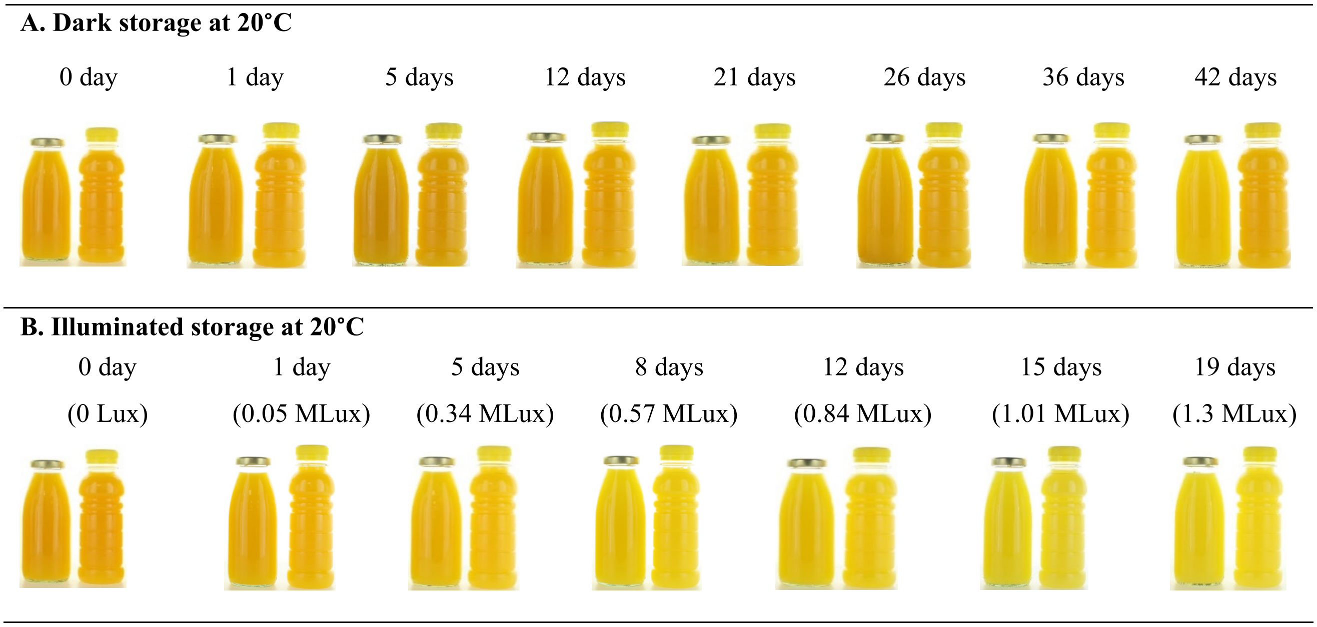

Figure S1. Model beverage samples during storage at 20°C in both (**A**) dark and (**B**) illuminated conditions.

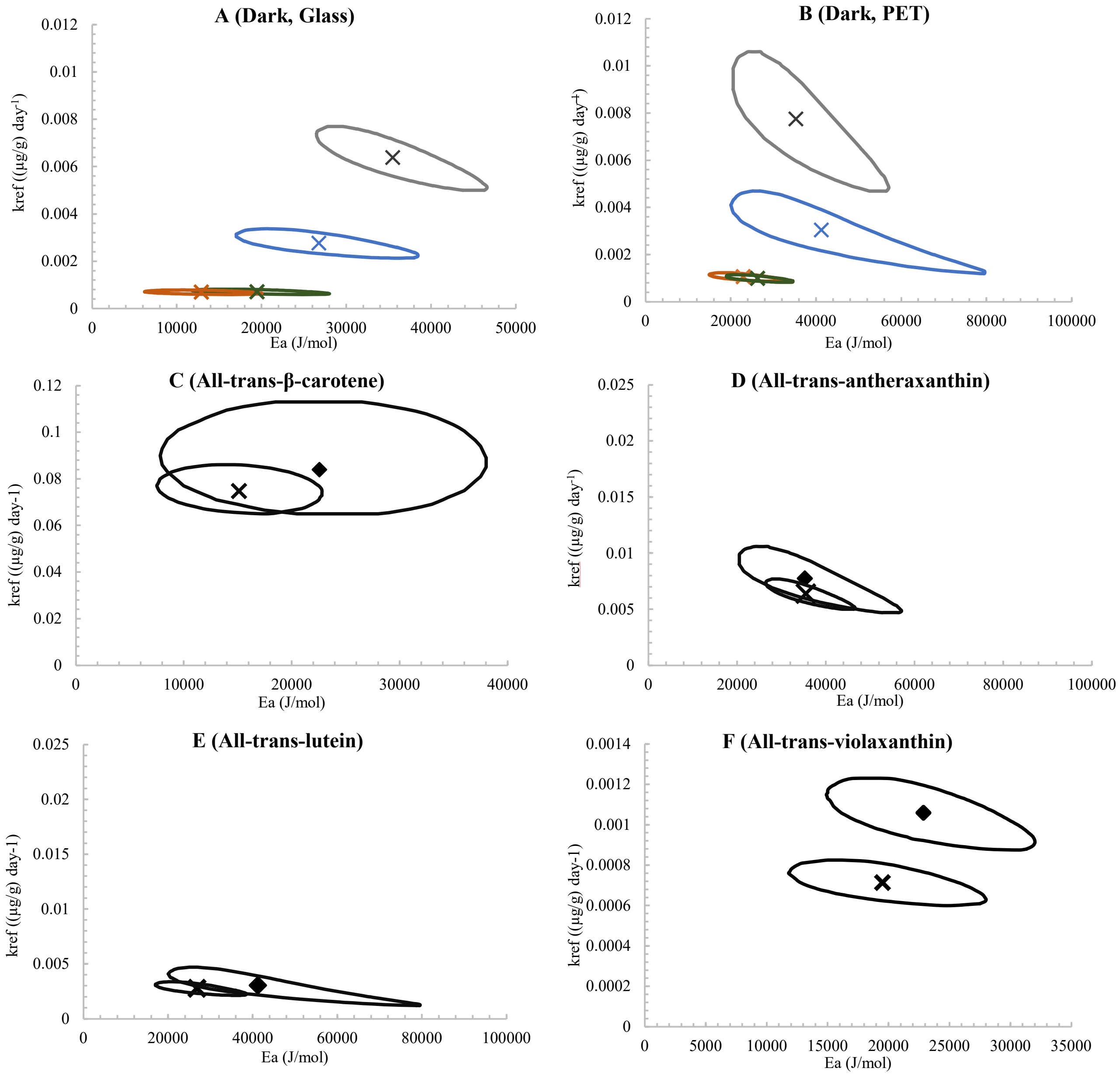

Figure S2. 90% Joint confidence regions of the estimated kinetic parameters (k_{ref} and Ea) for the changes in all-trans-carotenoid which were modelled using the same kinetic model during dark storage in (**A**) glass bottles and (**B**) PET bottles. ▬ All-trans-lutein; ▬ All-trans-antheraxanthin; ▬ All-trans-violaxanthin; and ▬ All-trans-neoxanthin. Graphs (**C**–**F**) illustrate the impact of packaging material used (◆ PET and ✖glass bottles) on the k_{ref} and Ea of each all-trans-carotenoid studied during dark storage.

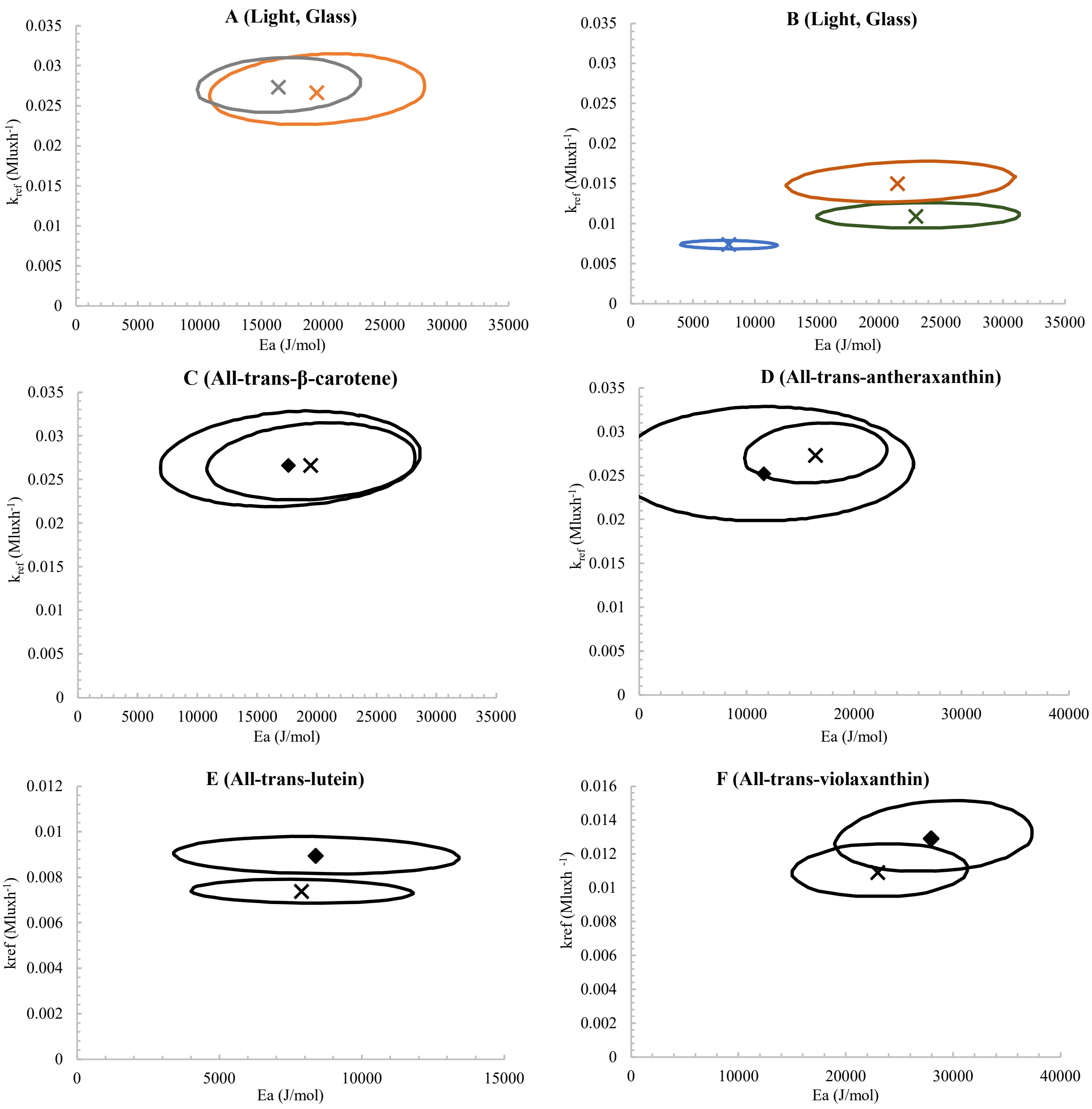

Figure S3. 90% Joint confidence regions (JCR) of the estimated kinetic parameters (k_{ref} and Ea) for the changes in all-trans-carotenoid which fitted to similar kinetic order models during illuminated storage in glass bottles. (**A**) All-trans-β-carotene and All-trans-antheraxanthin; and (**B**) All-trans-lutein, All-trans-violaxanthin, and All-trans-neoxanthin. Graphs (**C**–**F**) illustrate the impact of packaging material used (◆PET and ✖glass bottles) on the k_{ref} and Ea of each all-trans-carotenoid during illuminated storage.

Table S1. Chemical structure and absorption maxima of all-trans-carotenoids studied in the pumpkin juice concentrate-coloured beverage.

**Carotenoid**	**Structure**	**Absorption maxima (nm)[a]**			
All-trans-neoxanthin	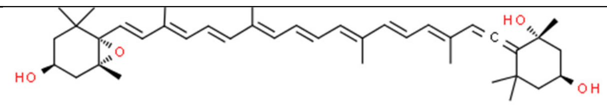	416	440	469	
All-trans-violaxanthin	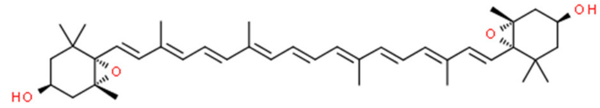		415	439	467
All-trans-antheraxanthin	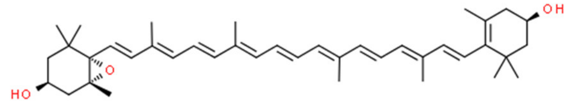	376	399	422	449
All-trans-lutein	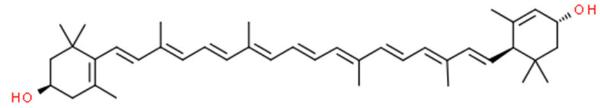		423	444	472
All-trans-β-carotene	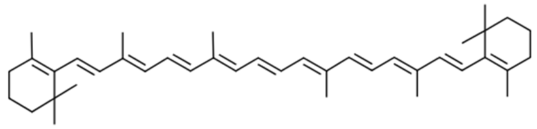		422	450	478

[a] Carotenoid extracts dissolved in methanol:acetone (2:1 v/v).
